# Unusual CT perfusion imaging pattern with normal CT angiography due to unintended intra-arterial contrast injection; report of 2 cases

**DOI:** 10.1016/j.radcr.2021.04.002

**Published:** 2021-05-05

**Authors:** Shatha Al Hilli, Vinu Mathew, Zeyad Tareq Jaleel

**Affiliations:** Department of Radiology, Hamad General Hospital, Doha, 3050 Qatar

**Keywords:** Intraarterial injection, CT perfusion artifacts, Contrast media administration, Intraarterial cannulation

## Abstract

Suspected stroke patients that arrive to the emergency department often start with non–contrast CT head followed immediately by CT perfusion and CT angiography, depending on the clinical suspicion and urgency. We present two cases of a 41-year-old male and 37-year-old female with unusual findings on the CT perfusion andnormal CT angiography study due to unintended intraarterial placement of intravenous cannula. This can give rise to unusual imaging pattern and thus awareness of this possibility can mitigate the diagnostic challenge that it brings up.

## Introduction

The stroke protocol in our institution includes a non–enhanced CT head followed by CT perfusion and lastly the CT angiography. The non–enhanced CT head is reviewed for any bleed or obvious infarct. The decision is then made to proceed for CT perfusion study and CT angiography. The perfusion study starts with the power injection of 40 mL of contrast at a rate of 8 ml/sec, followed by CT angiography that is done via bolus tracking at the descending aorta with 80 mL contrast at 4 mL/sec. All images were acquired by Somatom Definition Drive 256 slice CT scanner with dual source and dual energy and analysis of the data was performed on a separately connected syngovia workstation.

## First case

**Fig. 1:**
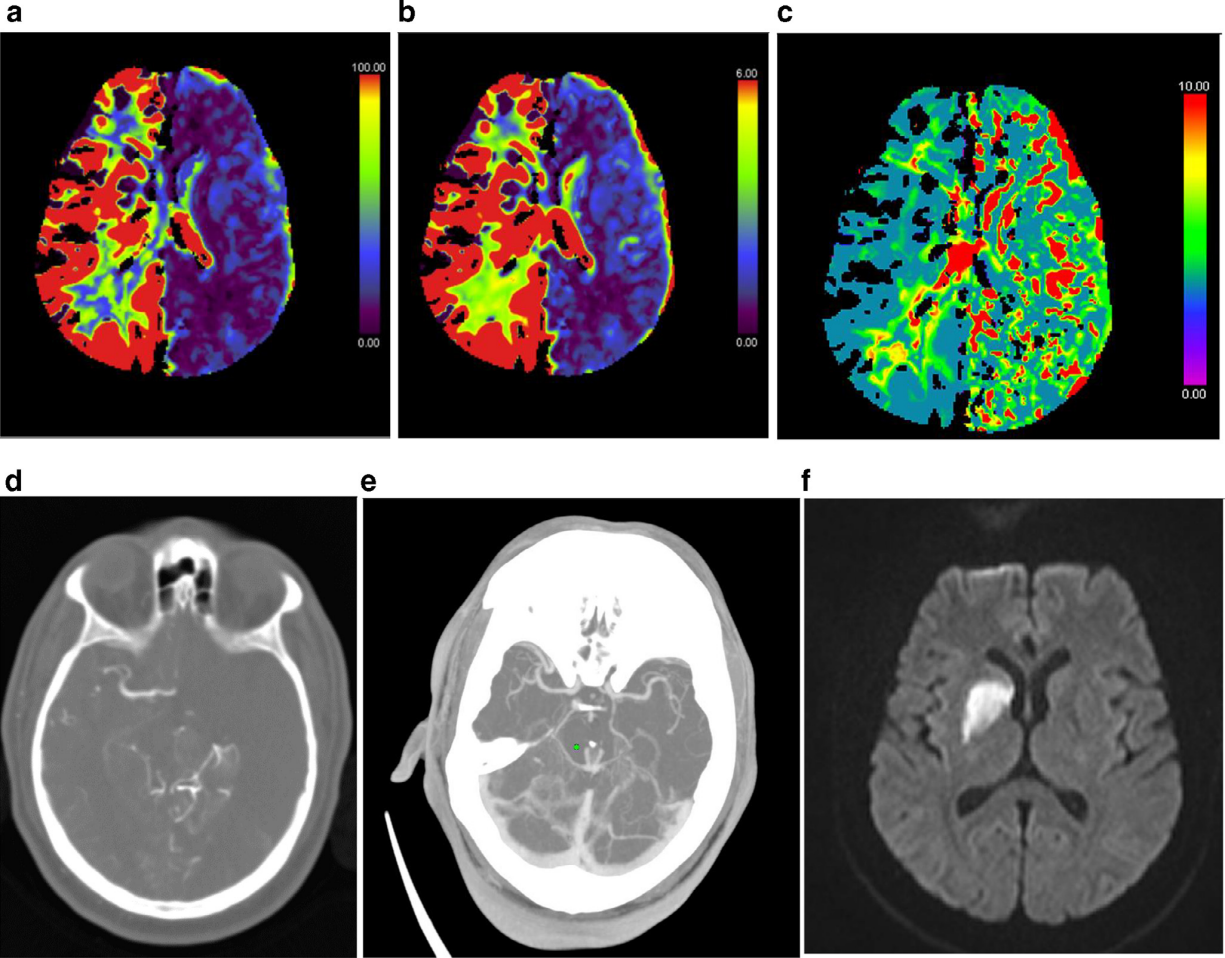
(A): Cerebral Blood Flow (CBF) in CT perfusion showing no flow in the in the left cerebral hemisphere with normal flow in the right cerebral hemisphere. (B): Cerebral Blood volume (CBV) in CT perfusion showing no volume the in the left cerebral hemisphere with normal volume in the right cerebral hemisphere. (C): CT perfusion (Mean Transit Time MTT) shows increased transit time in the left cerebral hemisphere. (D): CT perfusion Raw images (volume perfusion computed tomography (VPCT) DynMulti4D)- showing flow seen in the right middle cerebral arteries with absent left middle cerebral artery circulation. (E): CT angiography maximum intensity projection (MIP) images: Show normal flow in both middle cerebral artery territories. (F): MRI, Diffusion weighted images show diffusion restriction in the right basal ganglia region with no corresponding diffusion restriction in the left cerebral hemisphere.

First case was a 41-year-old male with history of hypertension. He presented with dysarthria and left sided weakness and presented to our institution by 1 PM. A neurologist reviewed the patient and requested the stroke protocol for this patient as he was within the window period. Plain study showed no obvious intracranial hemorrhage or obvious infarct. CT perfusion was done for the patient with Cerebral Blood Volume (CBV; Fig. 1A) and Cerebral Blood Flow (CBF; Fig. 1B), showed no flow in the left cerebral hemisphere and normal flow in the right cerebral hemisphere. Mean transit time (MTT; Fig. 1C) shows increased transit time in the left cerebral hemisphere. Raw CT perfusion images (Fig. 1D) confirmed the findings seen in the previous images. But CT angiography showed normal opacification of the arterial systems (Fig. 1E). MRI that was done next day showed no corresponding diffusion restriction in the left cerebral hemisphere (Fig. 1F) with diffusion restriction seen in the right basal ganglia.

## Second case

**Fig. 2:**
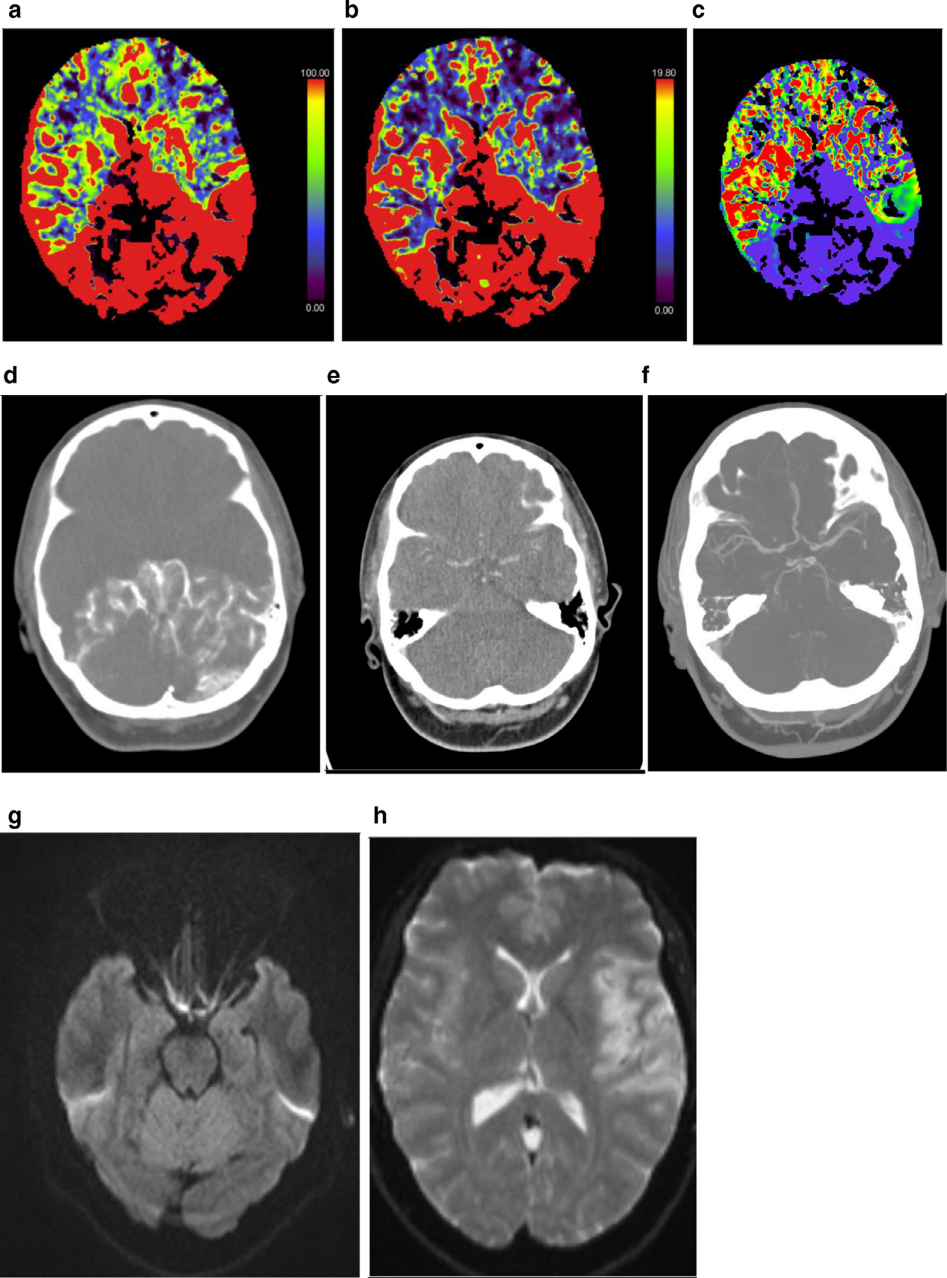
(A) CBF in CT perfusion showing increased flow in the posterior circulation arteries. (B): CBV in CT perfusion showing increased blood volume in the posterior circulation arteries. (C): CT perfusion (MTT) shows decreased transit time in the posterior circulation arteries. (D): CT perfusion early Raw images (VPCT DynMulti4D) – showing increased flow seen in the posterior circulation arteries with absent flow in the anterior circulation in the initial raw images. (E): CT perfusion late raw images showing delayed enhancement of the both anterior and posterior circulation. (F): CT angiography MIP images: Show normal flow in anterior and posterior circulation. (G): MRI, Diffusion weighted images showed no diffusion restriction. (H). MRI, Diffusion weighted images showing diffusion restriction in the left temporo-parietal region.

Second case was a 37-year-old female with no past medical history. She presented with right facial weakness and presented to the hospital around midnight. Stroke protocol was activated by the neurologist as the patient was within the window period. Plain study showed no obvious intracranial hemorrhage or obvious infarct. CT perfusion was done with CBV (Fig. 2A) and CBF (Fig. 2B) that showed increased predominant flow in the posterior circulation regions with early raw CT perfusion images (Fig. 2D) confirmed the findings seen in the previous images. The late raw CT perfusion images (Fig. 2E) shows delayed enhancement of anterior circulation arterial system. Mean transit time (MTT; Fig. 1C) shows decreased transit time in the posterior circulation arteries. CT angiography showed normal opacification of the arterial systems (Fig. 2F). MRI done on the same day (Fig. 2G) showed no obvious diffusion restriction in the posterior circulation but (Fig. 2H) there was diffusion restriction seen in the left temporo-parietal region.

## Discussion

In both cases, when the perfusion images are first seen, it could represent occlusion of the arterial supply or even a matched infarcted region but with CT angiography showing normal flow the interpretation can create a diagnostic dilemma. Contrast injection is routinely given via intravenous approach with the catheter inserted usually in the superficial veins in the antecubital fossa. The strikingly confusing perfusion values seen in both cases can be explained if the cannula was placed into the arterial system unintentionally instead of the normal venous cannulation. Revision of arterial system anatomy**_1_** can help comprehend why these 2 cases of intraarterial injection of the contrast showed 2 different imaging outcomes on the CT perfusion.

In the first case, the cannula was placed in the right cubital fossa likely the brachial artery. During the CT perfusion study that uses a high flow rate, the contrast would proceed retrograde into the right subclavian artery, vertebral artery, and into the brachiocephalic trunk which would then supply the right common carotid artery. This subsequently gave adequate perfusion to both the vertebral basilar system and right middle cerebral and anterior cerebral arteries. If there was enough pressure, the contrast could also theoretically go further retrograde and reach the aorta, which would then have opacified the left common carotid and left subclavian artery giving potentially a normal CT perfusion study. He was managed conservatively as a case of right basal ganglia stroke and discharged 5 days later with follow up in stroke clinic in 2 weeks.

In the second case, the placement of the cannula was in the left cubital fossa likely the brachial artery, as described above the contrast would proceed go retrograde to the left subclavian artery, and subsequently the intracranial arteries via the left vertebral artery. Even if there was enough force to cause more retrograde flow into the aorta, there would be no opacification of the brachiocephalic trunk or the left common carotid artery. This is because the brachiocephalic trunk and left common carotid artery are both located to the right of the left subclavian artery anatomically and thus the flow of blood would push the contrast that comes from the left subclavian artery further distal into the descending aorta. She was managed conservatively as a case of left temporo-parietal stroke and discharged 4 days later with follow up in stroke clinic in 2 weeks.

The remaining question is why did the CT angiography following the CT perfusion study show normal findings? Our hypotheses are that since CT angiography uses a slower flow rate of 4 mL/sec vs 8 mL/sec of the CT perfusion study, it is possible that the slower flow rate does not cause the flow of contrast to flow retrograde**_2_** as seen in CT perfusion but rather following the normal arterial circulation distally to the arm and returning via the venous system. In both the CT angiogram done in our study shows the average contrast enhancement of the subclavian vein (HU 268) is significantly lower than the average contrast enhancement seen in the normal CT angiography studies (HU 1206). This significantly decreased contrast enhancement of the subclavian vein in our studies may suggest our hypotheses that the contrast is following the normal arterial circulation distally to the arm and returning via the venous system. When returning via the venous system, the contrast will now opacify the cerebral arteries normally, thus resulting in normal CT angiography studies. This can be confirmed by checking the source time density curve and seeing the delayed time to peak of contrast in comparison to normal studies that could not be recovered in our cases. In the second case the early raw CT perfusion shows flow in the posterior circulation only and subsequent late raw CT perfusion images showing enhancement of the arterial system that may be explained that though most contrast proceeded retrograde fashion, some of the contrast proceeded as described via the venous circulation in the arm and caused delayed arterial enhancement.

## Conclusion

Intraarterial placement of intravenous cannula can give rise to unusual pattern of CT perfusion and CT angiography studies and thus awareness of this possibility can mitigate the diagnostic challenge that it brings up.

## Patient consent

No consent was taken as the images are entirely anonymized along with non–identifiable information.

